# Mechanisms of Social Media Effects on Attitudes Toward E-Cigarette Use: Motivations, Mediators, and Moderators in a National Survey of Adolescents

**DOI:** 10.2196/14303

**Published:** 2019-06-27

**Authors:** Hyunyi Cho, Wenbo Li, Lijiang Shen, Julie Cannon

**Affiliations:** 1 School of Communication The Ohio State University Columbus, OH United States; 2 Department of Communication Arts and Sciences Pennsylvania State University University Park, PA United States; 3 Department of Communication Cornell University Ithaca, NY United States

**Keywords:** adolescents, e-cigarettes, motivation, affordances, agency, realism, self-expression, social comparison, social learning, social media, filter, uses and gratifications

## Abstract

**Background:**

Exposure to risk behavior on social media is associated with risk behavior tendencies among adolescents, but research on the mechanisms underlying the effects of social media exposure is sparse.

**Objective:**

This study aimed to investigate the motivations of social media use and the mediating and moderating mechanisms of their effects on attitude toward electronic cigarette (e-cigarette) use among adolescents.

**Methods:**

Using data from a national sample survey of adolescents (age=14-17 years, N=594), we developed and validated a social media use motivation scale. We examined the roles of motivations in the effect of social media use on risk exposure and risk attitude.

**Results:**

Motivations for social media use included agency, self-expression, realism, social learning, social comparison, and filter. These motivations were associated differentially with the frequency of use of Facebook, Instagram, Snapchat, and YouTube. Frequency of social media use was positively associated with exposure to e-cigarette messages across the four platforms (*P*s<.001). Exposure to e-cigarette messages on Instagram (*P*=.005) and Snapchat (*P*=.03) was positively associated with attitude toward e-cigarette use. Perceived social media realism moderated the effects of e-cigarette message exposure such that when realism was high, the exposure effect was amplified, but when realism was low, the effect was mitigated (*P*<.001). A three-way interaction effect (*P*=.02) among exposure, social learning motivation, and social norm on attitude toward e-cigarette use was found. When perceived social norm was high, the moderating effect of social learning motivation on e-cigarette use attitude was amplified, but when social norm was low, the social learning motivation effect was attenuated.

**Conclusions:**

Because perceived social media realism moderates the effect of exposure to e-cigarette messages on attitude toward e-cigarette use, future intervention efforts should address the realism perceptions. The three-way interaction among exposure, social learning motivation, and social norm indicates the importance of addressing both the online and offline social environments of adolescents. The social media use motivation scale, reflecting perceived affordances, is broadly applicable. Understanding social media use motivations is important, as they indirectly influence attitude toward e-cigarette use via frequency of social media use and/or frequency of exposure to e-cigarette messages on social media.

## Introduction

### Background

Exposure to risk-related content on social media has been associated with positive attitudes toward risk behavior and adoption of the risk behavior, including alcohol use [1] and drug use [2]. Research shows that adolescents are particularly vulnerable to such social media influences, as the generation is growing up with social media as an important source and channel of socialization and as a social environment [3]. According to a Pew Research Center survey [4], the most popular platforms among adolescents were YouTube, followed by Instagram, Snapchat, and Facebook.

A particularly concerning risk behavior among adolescents is e-cigarette use. Although recent research shows that e-cigarette use is more efficacious than nicotine-replacement products in helping cigarette smokers quit [5], uptake of e-cigarette use can be harmful for adolescents. Research has found that e-cigarettes contain nicotine [6], which adversely affects brain development up to the age of 25 years [7]. Nicotine use during adolescence elevates the risk of addiction to other drugs [8]. Furthermore, the aerosol from e-cigarettes exposes both users and bystanders to harmful substances [9]. For these reasons, in 2018, the US Surgeon General called for urgent and aggressive action to protect young people from the harm, declaring youth e-cigarette use an epidemic [10].

Although findings connecting social media exposure and risk behavior including e-cigarette use have been increasing, sparse research has investigated the mechanisms with which the effects occur. The goal of this study was to address this gap in knowledge by investigating *how* social media influences adolescent e-cigarette use. When the processes underlying the connection between exposure and effect are identified, strategies for effective interventions could be developed. Therefore, this study sought to identify motivations of social media use and to investigate their mediating and moderating roles in the association between social media–based risk exposure and risk behavior attitude among adolescents.

### Conceptual Bases

In this study, we conceptualize social media use as a motivated behavior driven by individuals’ desire to fulfill their psychological and social needs [11] and hypothesize that differential motivations influence individuals’ choice of differential social media platforms, as they offer differential affordances. Frequency of use of differential platforms will be differentially associated with e-cigarette message exposure, which, in turn, influences attitude toward e-cigarette use.

To understand the effects of social media use on risk behavior attitudes, the motivations for social media use should be examined first. The uses and gratifications framework [11] explains that people use media to fulfill their individual needs and that there are motivations associated with people’s choice to consume media. For a given medium use (eg, television), there can be a range of motivations. Television use motivations, for example, include learning, relaxation, companionship, escape, arousal, and passing time [12,13]. Building on the uses and gratifications paradigm, scholars have advanced the uses and effects perspective, which predicts that differential motivations of media use will lead to differential effects [13,14]. Despite the theoretical advance, little research has empirically examined the relationship between motivations and effects, especially with respect to motivations, uses, and effects of social media.

As the media landscape has changed from mass media including television to the internet to Web 2.0, researchers have investigated the motivations associated with the use of various social media platforms. Studies have examined motivations or gratifications associated with the use of Facebook [15], Twitter [16], Instagram [17], YouTube [18], and Snapchat [19]. Although the information about motivations of using each social media platform is valuable, as they may be variable across the platforms, the motivations may also share commonalities. It may be more advantageous to study new media as a mix of attributes rather than discrete entities [20], as identifying the core motivations that can be differentially applied to different platforms may be of greater generative utility than identifying platform-specific motivations.

Moreover, we focus on the unique characteristics of Web 2.0 in this research. The existing conceptualizations and measurement of social media use motivations draw on the typology that has been used for traditional mass media use [21]. Observing that the available descriptions of new media gratifications may be more general than the nuanced gratifications available through new media, Sundar and Limperos [21] suggested possible new gratifications from new media, anchoring them to four classes of features: modality, agency, interactivity, and navigability.

Finally, extant research has yet to take into account the aspects of user-generated content. In mass media, users did not have a chance to contribute content, as the mass media content has been created by professionals. In contrast, Web 2.0, on which various social media platforms are based, relies on user-generated content and collective knowledge and sentiment [22,23]. Three interdependent gratifications available from user-generated media include consumption, participation, and production [22]. This study seeks to capture this new media ecology available on Web 2.0.

Moreno and colleagues’ [24] Facebook influence model captures a number of new gratifications linked to Facebook use, some of which could be extended to other social media use. Through a conceptual mapping approach, they identified 10 facets of Facebook use gratifications, including connection to people, far reaching, fast communication, curiosity about others, business and promotion, accessibility/adaptability, data/information, social norm establishment, identity expression, influence on identity, distraction, positive experience, and negative experience [24].

### Motivations for Social Media Use

Based on the past research reviewed above, we aimed to identify core motivations that may underlie the use of divergent social media platforms and may explain the social and psychological processes of social media effects. These motivations included agency, self-expression, realism, social learning, social comparison, and filter. The attributes and features available on various social media platforms may differentially accommodate these motivations. Notably, as discussed below, the social aspects of the motivations are frequently networked in nature, and the psychological motivations contribute to collective intelligence and sentiment on Web 2.0.

The agency and self-expression motivations represent the identity-integral function of social media. Not included in motivations ascertained for the use of mass media, the abovementioned motivations indicate a unique aspect of Web 2.0, which is often characterized as participative and user-generated. Agency refers to the motivation to influence others by sharing one’s own ideas and messages. Relevant to the agency motivation, research found that the expressive and performative involvement in user-generated content on the internet facilitated online and offline political participation among adolescents [25,26]. Agency was included as a gratification integral to new media uses [21] and Facebook use [24].

The self-expression motivation reflects a gratification sought and obtainable from user-generated media, unavailable from mass media. On user-generated media, the desire for production intersects with the desire for participation and consumption [22]. The internet makes it possible for individuals to experiment with their identities [27], and youth express their identities on social media while concurrently being open to social media’s influences on these identities [24]. Creativity was one of the motivations associated with young adults’ use of Instagram [17].

Perceived media realism has been an important explanatory variable of media effects [28]. In this study, however, we conceptualize social media realism differently from previous research on mass media realism. With traditional media, the industry and professionals determined the content. In social media, users are creators of the content as well as its consumers. Therefore, we developed items to capture this unique aspect of social media content and users’ assessment of the content created by other users.

The media provide a powerful apparatus to learn about the world and others. Social learning has been an important motivation for using media [12,13]. Mass media research found, for example, that soap opera viewers with information motivation reported greater attention to the program and engagement with characters during viewing than viewers with the motivation to merely pass time [29]. Social learning motivation has been linked to the use of Facebook [30] and Instagram [17]. Of note, in this study, we assert that the “social” in social learning motivation for using social media differs from the motivation for using mass media. In social media, the “social” is frequently networked in nature. On the other hand, in mass media, the learning motivation is more societal than social, as the target of learning is more diffusive in nature.

With metrics such as likes, ratings, and positive and negative comments that were unavailable in mass media, social media provides insight on others’ behaviors and attitudes. With these features, social media may provide knowledge about social norms [21,24,31] and a benchmark for social comparison [32]. Adolescents’ social comparison activities in social media affect their identity development under certain conditions [33].

In addition, social media allows users to construct their own world by using the function of filters [21,34], which may provide psychological contentment and comfort functions but could also obstruct a more comprehensive and objective perspective on what is going on in society [35]. As in this virtual community, only like-mined people may congregate together, reinforcing and strengthening shared viewpoints, concerns have been raised that social media filters can facilitate social fragmentation and polarization [36,37].

With regard to the motivation mechanisms, we propose the following hypothesis:

Hypothesis 1: Social media motivations include agency, self-expression, perceived realism, social learning, social comparison, and filter.

### Mediating Mechanisms

The abovementioned motivations may reflect perceived affordances of social media. Affordances are “action possibilities” toward which the stimuli in the environment suggest that humans act [38]. The concept of perceived affordances refers to user experiences and evaluations rather than the features themselves [39]. As each social media platform offers a mix of features, use of these platforms comes with convergent and divergent gratifications. For example, some of the Facebook use gratifications (eg, identity expression) [24] overlap with the Instagram use gratifications (eg, creativity) [17]. Communication channels including phone, email, texting, Facebook, and Snapchat were perceived to provide differential levels of social affordances [40]. Therefore, the mix of motivations is likely associated differentially with the use of differential social media platforms. The frequency of using social media, in turn, may predict the probability of exposure to e-cigarette advertisements and posts. At present, social media is the main outlet of e-cigarette marketing activities [41-43]. Social media–based exposure to e-cigarette messages was positively associated with e-cigarette expectancies among young adults [44]. Extending this prior research, we aim to investigate the association between social media e-cigarette exposure and attitudes toward e-cigarette use in a population of adolescents. Furthermore, we seek to examine the process of the exposure effects.

With regard to the mediating mechanisms, we propose the following hypotheses:

Hypothesis 2a: Social media use motivations are differentially associated with the frequency of use of differential social media platforms.

Hypothesis 2b: Social media use frequency is positively associated with exposure to e-cigarette messages on social media.

Hypothesis 2c: Exposure to e-cigarette messages on social media is positively associated with attitude toward e-cigarette use.

### Moderating Mechanisms

#### Two-Way Interaction

In addition to indirectly influencing e-cigarette message exposure and e-cigarette use attitude, social media use motivations may moderate the effect of the exposure on attitude, that is, the effects of risk exposure on social media can be attenuated or amplified depending on the motivations of social media use. One of the factors that could modulate harmful social media effects may be realism judgment. The more users believe that other user-generated content is a representation of their true self and a truthful depiction of their beliefs, emotions, and lives, the stronger are the effects of the exposure to the content. On the other hand, if the users think that social media representations deviate from users’ true selves and lives, the effects of the exposure would be mitigated.

#### Three-Way Interaction

The social dimensions of social media use motivation (eg, social learning, social comparison, and filter) may also moderate the effects of risk exposure on social media. Importantly, because of the networked nature of the social world represented in one’s social media environment, the influence of the social motivations may be qualified by social norm. For example, while those who use social media with stronger social learning motivation are more likely to be affected by prevalent or glamorous depictions of risk behavior than those with weaker social learning motivation, the influence of social learning motivation may be further moderated by the kind of social world that one maintains. If, for example, friends in one’s social network use e-cigarettes, then the effect of social learning motivation may be amplified. However, if no one in one’s social network uses e-cigarettes, then the effect of social learning motivation may be mitigated.

Regarding the moderating mechanisms, we proposed the following hypotheses:

Hypothesis 3a: The effect of exposure on attitude is moderated by realism motivation such that high realism amplifies the exposure effect on attitude and low realism attenuates it.

Hypotheses 3b-d: The effect of exposure on attitude is moderated by social learning (b), social comparison (c), and filter motivations (d), which are, in turn, moderated by social norm. When social norm is high, the moderating effects of these motivations on attitude are amplified, but when social norm is low, the effects of these motivations are attenuated.

## Methods

### Design

This study was conducted as part of a larger project investigating adolescent social media use and risk behavior. Participants answered questions about their social media use, risk exposure on social media, and attitude toward e-cigarette use prior to seeing a 1-minute anti–e-cigarette video message. The items assessing motivations associated with social media use were given at the end of the study. To prevent exposure to the 1-minute video message from having any effect on the motivation measures, a battery of items, including those about other adolescent risk behaviors, was assessed after the exposure prior to the motivation assessment.

### Participants

Participants (N=594) were adolescents aged 14-17 (mean 15.48, SD 1.12) years recruited through Ipsos (formerly GfK), a survey firm providing a probability-based sample of the US population. Male and female adolescents comprised 47.4% and 52.6% of the sample, respectively. The majority of the adolescents were white individuals (65.0%); the rest were Hispanic (16.4%), black (7.6%), other (6.4%), and mixed race (4.6%) individuals. Both parental consent and adolescent assent were obtained prior to the study.

### Measures

Social media use motivations were measured using the scale designed for this study. Items were developed to measure the posited six dimensions of social media use motivations. They were a mixture of items taken from Moreno et al [24] and Sundar and Limperos [21] and those that were developed in this study. For the dimensions of agency, social comparison, and filter, items were taken from the studies of Moreno et al [24] and Sundar and Limperos [21]. Items for the self-expression dimension were taken from Moreno et al [24]. One item of the realism dimension was taken from Sundar and Limperos [21]. Multimedia Appendix 1 presents a full description of the social media use motivation measures. The response scale ranged from 1 - “strongly disagree” to 5 - “strongly agree.” Table 1 presents means, SDs, and reliability alphas of and correlations between the dimensions.

Regarding social media use, the frequency of use of the four most popular social media platforms among adolescents [4] was assessed. These platforms included Facebook, Instagram, Snapchat, and YouTube. The response scale ranged from 1 - “I don’t use this” to 7 - “five or more times a day.” Table 2 presents the descriptive data.

Exposure to e-cigarette messages on social media was measured by asking adolescents how often they saw (1) e-cigarette advertisements and (2) pictures in which people were using e-cigarettes on each of the aforementioned four social media platforms. These two items assessing exposure to ads and usage images were averaged to create an index for each platform. The two items were substantially correlated (r=0.75, 0.66, 0.63, and 0.65 for Facebook, Instagram, Snapchat, and YouTube, respectively). The response scale ranged from 1 - “never” to 5 - “very often.” Table 2 shows the distribution.

Attitude toward e-cigarette use was measured using the Osgood semantic differential scale [45]. Specifically, three pairs of bipolar adjectives including bad/good, undesirable/desirable, and unfavorable/favorable were provided and scored on a 5-point scale. Higher scores indicated more positive attitude.

The social norm measure was focused on friends’ descriptive norm and measured using the Fishbein scale [46] with the question “how many of your friends use e-cigarettes?” The response scale ranged from 1 - “none” to 4 - “all of them.”

**Table 1:** Means, SDs, reliability (α), and correlations (r) between social media motivations.

#	Motivation	Mean (SD)	α	r					
				1	2	3	4	5	6
1	Agency	3.29 (1.00)	0.94						
2	Filter	3.14 (0.81)	0.79	0.62					
3	Self-expression	3.13 (0.97)	0.91	0.78	0.58				
4	Social learning	3.41 (0.86)	0.91	0.68	0.71	0.61			
5	Social comparison	3.21 (0.94)	0.90	0.61	0.70	0.54	0.75		
6	Realism	2.69 (0.81)	0.84	0.52	0.60	0.54	0.49	0.50	

**Table 2:** Social media use and exposure to e-cigarette messages on social media among adolescents. All values are given as mean (SD) scores.

Factor	Facebook	Instagram	Snapchat	YouTube
General social media use	2.80 (2.20)	4.42 (2.53)	4.24 (2.63)	5.39 (1.80)
Exposure to e-cigarette messages	1.46 (0.77)	1.81 (0.89)	1.81 (0.93)	1.98 (0.92)

## Results

### Analysis Strategy

We employed confirmatory factor analyses to test hypothesis 1. Linear regression, PROCESS macro of Hayes [47], and bootstrapping estimation approach of Shrout and Bolger [48] were used to test hypotheses 2 and 3.

### Hypothesis 1

Hypothesis 1 predicted that social media motivations would include the dimensions of agency, self-expression, realism, social learning, social comparison, and filter. Confirmatory factor analyses were conducted to test this hypothesis. Unless unidimensionality of a scale is established for the first-order factors, evidence in support of its unidimensionality should be obtained from two sources: (1) a first-order oblique multifactor model that should fit the data and the correlations among the first-order factors should be similar, and (2) statistical equivalence has to be established between the first-order multifactor model and a second-order single-factor model. The degree of freedom for the first-order single-factor model (20 items, 1 factor) was 170, that for the first-order multifactor model (20 items, 6 associated factors) was 164, and that for the second-order single-factor model was 166. With these parameters and assuming α=0.05, a sample size of 568 yielded statistical power of >0.99 when testing these factor models [49].

Input and model specifications are as follows. Individuals’ responses to the 20 social media use items were submitted to LISREL 8.80 (Scientific Software International, Inc, IL) for confirmatory factor analyses. First, a first-order single-factor model was estimated, where all 20 items were specified to load on one latent factor. Second, a first-order oblique six-factor model was estimated, where the 6 first factors were allowed to be associated with each other. Third, a second-order single-factor model was estimated, where the second-order factor loaded on the 6 first-order factors, which were not allowed to be correlated.

To evaluate the overall fit of the models to the data, four fit indices were considered. First, the goodness-of-fit index (GFI) produces values ranging from 0 to 1, with values in excess of 0.90 indicating good fit. Second, the comparative fit index (CFI) produces values ranging from 0 to 1, with values larger than 0.90 indicating good fit. Third, Browne and Cudeck [50] contend that values of the root mean square error of approximation (RMSEA) of 0.08 or lower indicate reasonable fit, although values of 0.06 or below should be preferred. Fourth, the Bayesian information criterion (BIC) is constructed such that negative values provide evidence of model fit, while positive BIC values suggest problematic model fit. Differences in BIC of 2 are thought to provide some evidence; ≥6, strong evidence; and ≥10, very strong evidence for the superiority of the model with a more negative BIC value over another model [51].

The first-order single-factor model did not fit the data; the values of this model are as follows: χ^2^_170_= 3147.97, *P*<.001, RMSEA=0.21, GFI=0.57, CFI=0.86, and BIC=2061.06. The first-order oblique six-factor model was a good fit to the data: χ^2^_155_=639.18, *P*<.001, RMSEA=0.08, GFI=0.90, CFI=0.98, and BIC=–351.83. Additional evidence was obtained from (1) the standardized factor loadings (the three factors had similar and reasonably high loadings on the indicators, ranging from 0.76 to 0.92 and similar within each factor) and (2) the substantive correlations among the six factors (ranging from 0.54 to 0.84), thereby providing clear indication of nonorthogonality.

The second-order single-factor model was nested within the oblique first-order six-factor model. It was not surprising that the second-order single-factor model yielded worse fit than the first-order six-factor model: χ^2^_9_= 211.69 and *P*<.001. However, given the exceptional statistical power (ie, >0.99) in model testing, we considered this discrepancy to be acceptable. The absolute indices showed that the second-order single-factor model was also a good fit to the data: χ^2^_164_=850.87, *P*<.001, RMSEA=0.08, GFI=0.90, and CFI=0.98, except for BIC=–197.68. The values for RMSEA, GFI, and CFI were almost identical between the second-order single-factor model and the first-order six-factor model. Together, these values indicated that the second-order single-factor model provided a plausible account of the data. However, the Chi-square test and BIC difference of 156.15 in favor of the first-order six-factor oblique model suggested potential variances in the factor structure of social media motivations.

**Figure 1:**
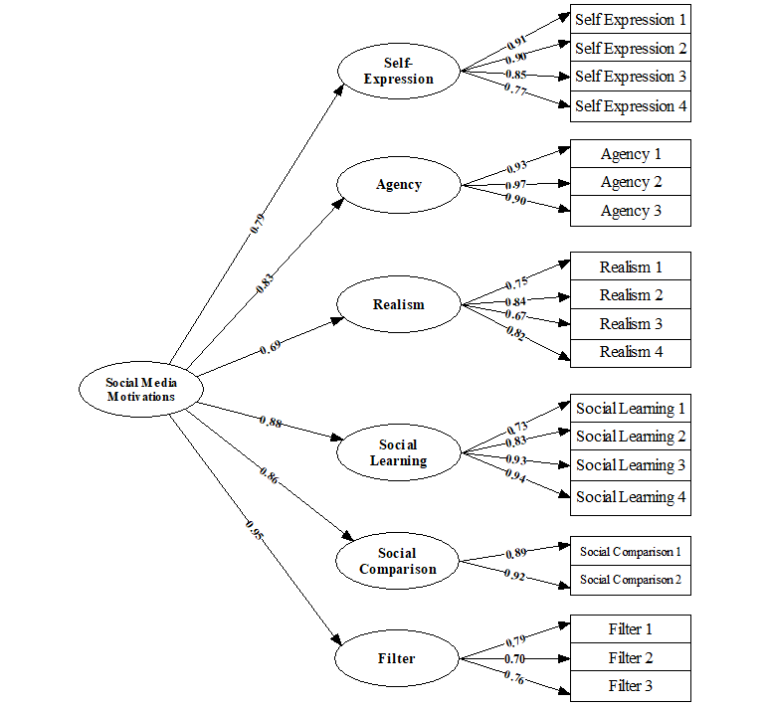
Factor structure.

The factor loadings of the six first-order factors on the second order factor provided additional support: The factor loadings were 0.78 for self-expression, 0.82 for agency, 0.68 for realism, 0.87 for social learning, 0.85 for social comparison, and 0.94 for filter. These results provided evidence that the second-order single-factor model was adequate for the social media use motivation scale and could be considered equivalent to the first-order six-factor model. Figure 1 presents the factor structure.

### Hypothesis 2

Hypothesis 2a predicted that social media use motivations would be associated differentially with different social media platform usage. For each of the four social media platforms, the frequency of use was regressed onto the six social media use motivations and the covariates of adolescent age, sex, and race. As Table 3 summarizes, the motivations were associated differentially across the four platforms. Facebook use was associated with the motivations of agency (*P*=.005) and filter (*P*=.008). Instagram use was associated with self-expression (*P*<.001) and social learning motivations (*P*=.009). Likewise, Snapchat use was associated with self-expression (*P*<.001) and social learning (*P*=.01). YouTube use was associated with social comparison motivation (*P*=.002). In this regression model, where the motivations were controlling for each other, realism was unrelated to any of the social media platform use. However, without controlling for other motivations (but controlling for age, sex, and race), realism was associated with the use of Facebook (β=0.209), Instagram (β=0.201), and Snapchat (β=0.177; all *P*<.001), but not YouTube (β=0.071, *P*=.09). Similarly, realism was correlated with the use of Facebook (r=0.210), Instagram (r=0.208), and Snapchat (r=0.185), but not YouTube (r=0.074, *P*=.07).

Hypothesis 2b predicted that social media use would be positively associated with exposure to e-cigarette messages on social media. For each social media platform, the frequency of exposure to e-cigarette messages was regressed on to the frequency of use of the platform while controlling for age, sex, race, and social media use motivations. Table 4 presents the results. Across the platforms, social media use was significantly associated with exposure to e-cigarette messages.

Hypothesis 2c predicted that exposure to e-cigarette messages on social media would be positively associated with attitude toward e-cigarette use, which was regressed onto age, sex, race, social media use motivations, frequencies of using social media, and frequencies of exposure to e-cigarette messages on social media. The results are presented in Table 5. Significant positive associations between e-cigarette message exposure on Instagram and Snapchat and attitude toward e-cigarette use were found. The more frequently adolescents saw e-cigarette messages on each of these platforms, the more positive were their attitudes toward e-cigarette use.

Serial mediation analyses using the PROCESS path-analysis macro developed by Hayes [48] were conducted to examine the overall predictions of Hypotheses 2a-c, such that the effects of social media use motivations on attitude toward e-cigarette would be mediated through frequency of social media use and exposure to e-cigarette information on each of the four social media platforms. A bootstrapping estimation approach with 5000 samples was used to test the indirect effects in each model [49].

Agency motivation increased attitude toward e-cigarette use through frequency of Facebook use, independent of exposure to e-cigarette information on Facebook (point estimate=0.02, 95% CI 0.001-0.049), but not through exposure to e-cigarette information on Facebook, independent of frequency of Facebook use (point estimate=–0.002, 95% CI –0.016 to 0.007), and not serially through frequency of Facebook use and exposure to e-cigarette information on Facebook (point estimate=0.004, 95% CI –0.006 to 0.018). There was no direct effect of agency motivation on attitude toward e-cigarette use (point estimate=–0.05, 95% CI –0.174 to 0.072).

**Table 3:** Associations between motivations and frequency of social media use among adolescents.

Motivation	Social media platform										
	Facebook			Instagram			Snapchat			YouTube	
	B	β	B		β	B		β	B		β
Agency	0.433	0.197^a^	–0.153		–0.060	–0.264		–0.100	–0.058		–0.031
Filter	0.478	0.177^a^	0.218		0.071	0.302		0.093	0.112		0.054
Self-express	–0.165	–0.075	0.680		0.258^b^	0.651		0.237^b^	0.010		0.000
Social learning	–0.190	–0.072	0.515		0.178^a^	0.518		0.171^c^	0.082		0.044
Social comparison	0.121	0.049	–0.184		–0.069	–0.311		–0.111	0.381		0.197^a^
Realism	0.138	0.054	–0.019		–0.004	0.042		0.014	–0.164		–0.070

^a^*P*<.01.

^b^*P*<.001.

^c^*P*<.05.

**Table 4:** Associations between social media use frequency and e-cigarette message exposure frequency among adolescents.

Social media platform	B	Standard error	β	*P* value
Facebook	0.165	0.013	0.471	<.001
Instagram	0.163	0.014	0.459	<.001
Snapchat	0.174	0.013	0.487	<.001
YouTube	0.142	0.021	0.273	<.001

**Table 5:** Associations between exposure to e-cigarette messages on social media and e-cigarette use attitude among adolescents.

Social media platform	B	Standard error	β	*P* value
Facebook	–0.082	0.067	–0.071	.23
Instagram	0.214	0.076	0.217	.005
Snapchat	0.154	0.069	0.163	.03
YouTube	–0.079	0.056	–0.083	.15

Filter motivation increased attitude toward e-cigarette use through frequency of Facebook use independent of exposure to e-cigarette information on Facebook (point estimate=0.02, 95% CI 0.0002-0.055), not through exposure to e-cigarette information on Facebook, independent of frequency of Facebook use (point estimate=0.003, 95% CI –0.007 to 0.016), and not serially through frequency of Facebook use and exposure to e-cigarette information on Facebook (point estimate=0.005, 95% CI –0.007 to 0.019). There was no direct effect of filter motivation on attitude toward e-cigarette use (point estimate =–0.01, 95% CI –0.154 to 0.134).

**Figure 2:**
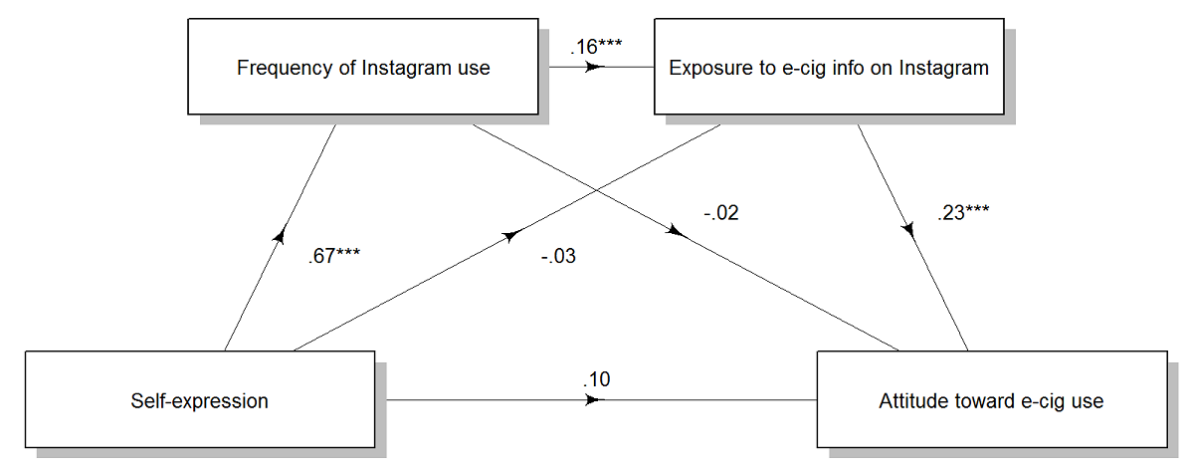
Effects of self-expression motivation on attitude toward e-cigarette use through frequency of Instagram use and exposure to e-cigarette messages on Instagram. Numbers are unstandardized regression coefficients. *P<.05, **P<.01, ***P<.001. e-cig/e-cigarette: electronic cigarette.

Self-expression motivation increased attitude toward e-cigarette use serially through frequency of Instagram use and exposure to e-cigarette information on Instagram (point estimate=0.03, 95% CI 0.009 to 0.048), not through frequency of Instagram use, independent of exposure to e-cigarette information on Instagram (point estimate=–0.01, 95% CI –0.039 to 0.011), or through exposure to e-cigarette information on Instagram, independent of frequency of Instagram use (point estimate=–0.01, 95% CI –0.039 to 0.024). There was no direct effect of self-expression motivation on attitude toward e-cigarette use (point estimate=0.10, 95% CI –0.019 to 0.222; Figure 2).

**Figure 3:**
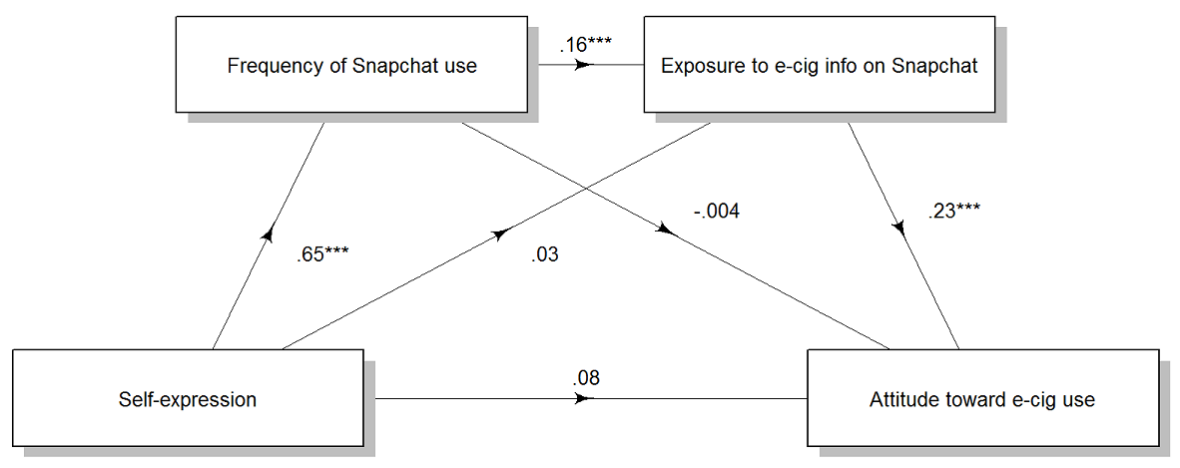
Effects of self-expression motivation on attitude toward e-cigarette use through frequency of Snapchat use and exposure to e-cigarette messages on Snapchat use. Numbers are unstandardized regression coefficients. *P<.05, **P<.01, ***P<.001. e-cig/e-cigarette: electronic cigarette.

Similarly, self-expression motivation increased attitude toward e-cigarette use serially through frequency of Snapchat use and exposure to e-cigarette information on Snapchat (point estimate=0.03, 95% CI 0.009 to 0.049), not through frequency of Snapchat use, independent of exposure to e-cigarette information on Snapchat (point estimate=–0.002, 95% CI –0.026 to 0.019), and not through exposure to e-cigarette information on Instagram, independent of frequency of Snapchat use (point estimate=–0.007, 95% CI –0.023 to 0.035). There was no direct effect of self-expression motivation on attitude toward e-cigarette use (point estimate=0.08, 95% CI –0.041 to 0.199; Figure 3).

**Figure 4:**
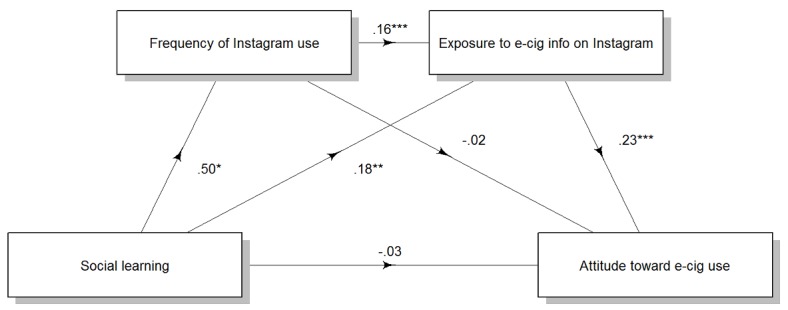
Effects of social learning motivation on attitude toward e-cigarette use through frequency of Snapchat use and exposure to e-cigarette messages on Snapchat. Numbers are unstandardized regression coefficients. *P<.05, **P<.01, ***P<.001. e-cig/e-cigarette: electronic cigarette.

Social learning motivation increased attitude toward e-cigarette use serially through frequency of Instagram use and exposure to e-cigarette information on Instagram (point estimate=0.02, 95% CI 0.003-0.040), through exposure to e-cigarette information on Instagram, independent of frequency of Instagram use (point estimate=0.04, 95% CI 0.012 to 0.085), but not through frequency of Instagram use, independent of exposure to e-cigarette information on Instagram (point estimate=–0.008, 95% CI –0.031 to 0.009). There was no direct effect of social learning motivation on attitude toward e-cigarette use (point estimate=–0.03, 95% CI –0.175 to 0.111; Figure 4).

**Figure 5:**
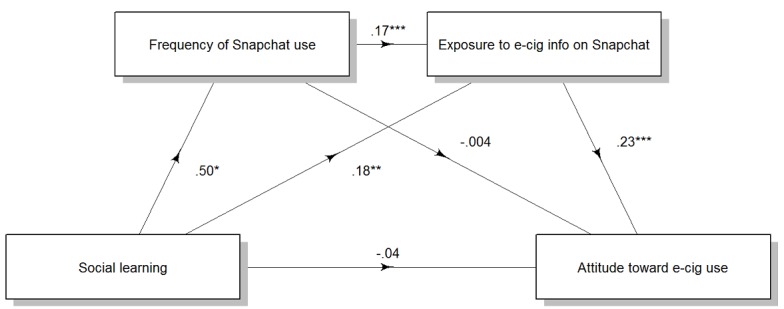
Effects of social learning motivation on attitude toward e-cigarette use through frequency of Instagram use and exposure to e-cigarette messages on Instagram use. Numbers are unstandardized regression coefficients. *P<.05, **P<.01, ***P<.001. e-cig/e-cigarette: electronic cigarette.

Similarly, social learning motivation increased attitude toward e-cigarette use serially through frequency of Snapchat use and exposure to e-cigarette information on Snapchat (point estimate=0.02, 95% CI 0.003-0.040), through exposure to e-cigarette information on Snapchat, independent of frequency of Snapchat use (point estimate=0.04, 95% CI 0.010-0.083), but not through frequency of Instagram use, independent of exposure to e-cigarette information on Instagram (point estimate=–0.002, 95% CI –0.021 to 0.016). There was no direct effect of social learning motivation on attitude toward e-cigarette use (point estimate=–0.04, 95% CI –0.183 to 0.103; Figure 5).

Lastly, the serial mediation from social comparison motivation to attitude toward e-cigarette use through frequency of YouTube use and exposure to e-cigarette information on YouTube was not significant. None of the paths in this model were significant.

### Hypothesis 3

Hypothesis 3a predicted that effects of social media–based exposure to e-cigarette messages will be differentially moderated by different social media use motivations such that high realism motivation will amplify the effect of the exposure, while low realism motivation will attenuate the effect. Moderation analyses using PROCESS macro with the 5000 bootstrapping estimation approach were conducted to examine the hypotheses [48,49].

**Figure 6:**
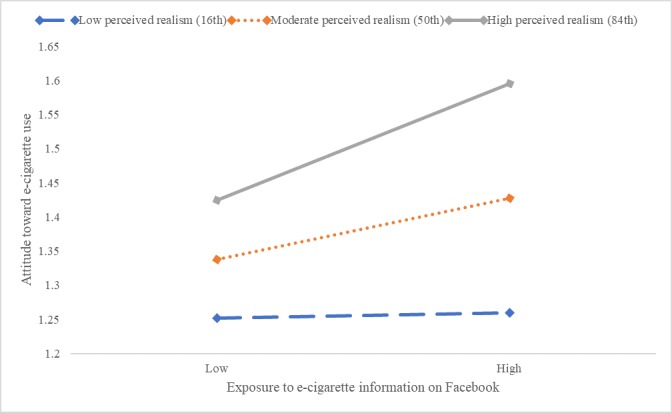
Moderating effects of perceived realism and exposure to e-cigarette information on Facebook on attitude toward e-cigarette use. e-cigarette: electronic cigarette.

The results showed that perceived realism interacted with exposure to e-cigarette messages on Facebook in influencing attitude toward e-cigarette use (*F*_1,562_=4.31, *P*=.04). Further inspection indicated that exposure to e-cigarette information on Facebook is related to attitude among adolescents with high perceived realism (point estimate=0.17, *P*=.001), not for those with moderate or low perceived realism (Figure 6).

**Figure 7:**
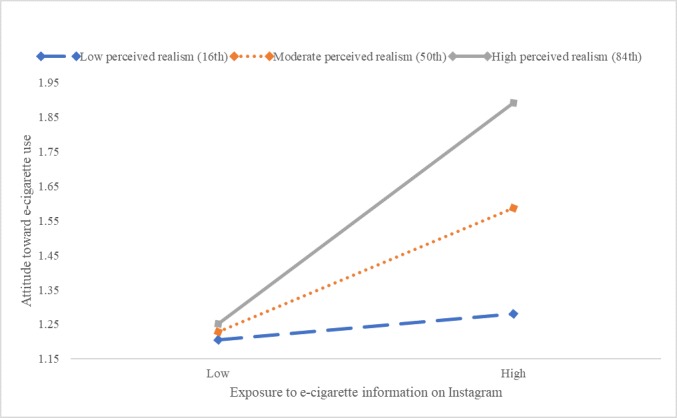
Moderating effects of perceived realism and exposure to e-cigarette information on Instagram on attitude toward e-cigarette use. e-cigarette: electronic cigarette.

Perceived realism also moderated the relationship between exposure to e-cigarette messages on Instagram and the attitude toward e-cigarette use (*F*_1,568_=15.88, *P*<.001). Further inspection indicated that exposure on Instagram was related to attitude among adolescents with moderate (point estimate=0.17, *P*<.001) and high perceived realism (point estimate=0.32, *P*<.001), not for those with low perceived realism (Figure 7).

**Figure 8:**
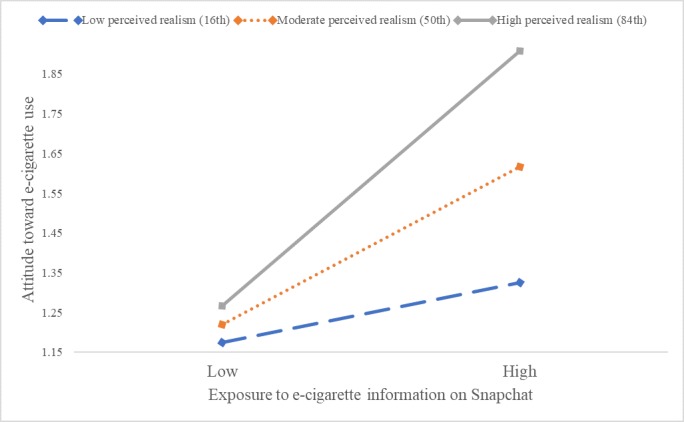
Moderating effects of perceived realism and exposure to e-cigarette information on Snapchat on attitude toward e-cigarette use. e-cigarette: electronic cigarette.

Similarly, perceived realism moderated the relationship between exposure to e-cigarette use on Snapchat and attitude toward e-cigarette use (*F*_1,566_=12.35, *P*<.001). This effect was observed only for adolescents with moderate (point estimate=.20, *P*<.001) and high realism (point estimate=0.32, *P*<.001), not for those with low realism (Figure 8).

**Figure 9:**
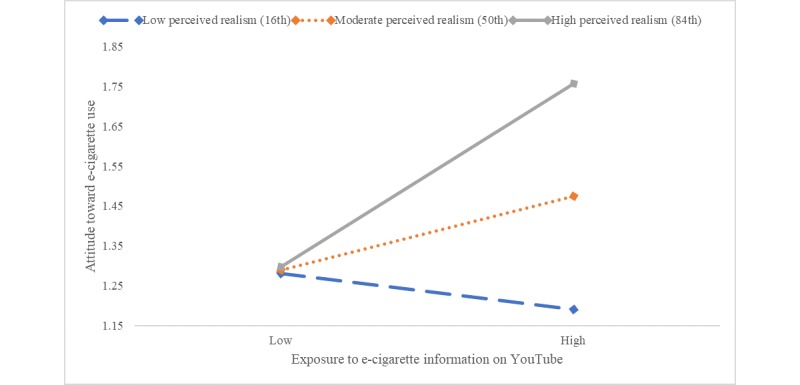
Moderating effects of perceived realism and exposure to e-cigarette information on YouTube on attitude toward e-cigarette use. e-cigarette: electronic cigarette.

Finally, another significant moderating effect was observed between perceived realism on exposure to e-cigarette messages on YouTube and the attitude toward e-cigarette use (*F*_1,569_=13.90, *P*<.001). The effect was observed only for adolescents with moderate (point estimate=0.09, *P*<.05) and high perceived realism (point estimate=0.23, *P*<.001), not for those with low perceived realism (Figure 9).

**Figure 10:**
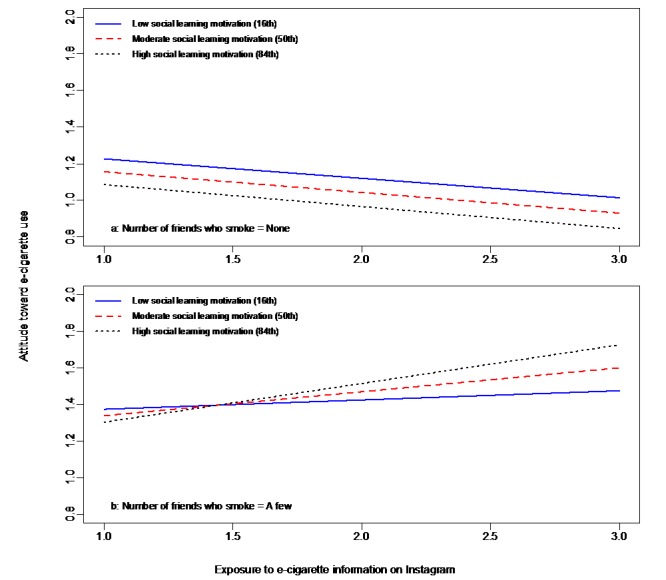
Effects of social norm on the moderating effects of social learning motivation on the relationship between exposure to e-cigarette information on Instagram and attitude toward e-cigarette use. e-cigarette: electronic cigarette.

Hypotheses 3b-d predicted three-way interactions among exposure, social motivations (ie, social learning, social comparison, and filter), and social norm. A significant three-way interaction among exposure on Instagram, social learning motivation, and social norm was found. As predicted, the moderating effect of social learning motivation on the relationship between exposure to e-cigarette information on Instagram and attitude toward e-cigarette use was found to depend on social norm (ie, the number of friends who use e-cigarettes; *F*_1,564_=5.14, *P*=.02). The three-way interaction effect was significant for those who had more than a few friends who used e-cigarettes but not for those without friends who used e-cigarettes. This indicates that for adolescents who had greater e-cigarette use norms in their networks, the higher the social learning motivation, the more positive their attitude toward e-cigarette use when they were exposed to more e-cigarette information on Instagram. The three-way interaction is depicted in Figure 10. Hypothesis 3b concerning social learning motivation was supported, while Hypothesis 3c-d regarding social comparison and filter motivations was not supported.

## Discussion

### Principal Findings

This study aimed at investigating the mechanisms of the effects of social media use on attitude toward e-cigarette use among adolescents. To this end, we first investigated motivations of social media use. Next, we examined the roles of motivations in the process of effects of social media use and e-cigarette message exposure on e-cigarette use attitude. Collectively, the results contribute to extant theoretical and practical knowledge about the mechanisms of social media effects on risk exposure and risk attitude.

In a survey of a national sample of 14- to 17-year-old adolescents, we found that their motivations for social media use included agency, self-expression, realism, social learning, social comparison, and filter (Hypothesis 1). To our knowledge, this is a first validated measure of social media use motivation that is not platform-specific, uses a mixed attributes approach, and recognizes the unique functions of Web 2.0. Furthermore, other results of this study demonstrate the utility of the scale and its components in understanding various social media use and their effects on risk exposure and attitude.

The six motivations predicted individual social media platform use differentially (Hypothesis 2a). Agency and filter motivations were associated with Facebook use, whereas self-expression and social learning were associated with Instagram and Snapchat use. Dissimilar to other platforms, YouTube use was associated with social comparison motivation.

The parsimoniousness of our social media use motivation scale can facilitate research and practice, as it provides an understanding of differential motivations attached to divergent social media platforms and thereby assisting efforts to examine their effects. Practically, as social media use motivations indirectly predicted attitude toward e-cigarette use through frequency of social media use and/or exposure to e-cigarette messages on social media (Hypothesis 2a-c), understanding these motivations and addressing them will be essential to future prevention efforts. Of note, the influences of self-expression and social learning motivations on attitude toward e-cigarette use were greater than those of other motivations, indicating the importance of addressing these two motivations.

Across the four popular social media platforms among adolescents, frequency of use significantly predicted frequency of exposure to e-cigarette messages (Hypothesis 2b). Although not all exposure predicted positive attitude toward e-cigarette use (Hypothesis 2c), the positive associations between frequencies of social media use and e-cigarette message exposure suggest the importance of addressing social media–based exposure to e-cigarette messages.

More frequent exposure to e-cigarette messages on Instagram and Snapchat predicted more positive attitude toward e-cigarette use among adolescents (Hypothesis 2c), suggesting that these two social media may require greater attention than other platforms from prevention efforts.

The role of YouTube observed in this study is noteworthy. Although adolescents’ volume of YouTube use was larger (Table 2), the association between exposure and attitude was not significant on YouTube (Hypothesis 2c). This may be because YouTube is a less networked platform than other social media such as Instagram and Snapchat. Exposure to e-cigarette messages on Facebook was lower than that on other social media, perhaps reflecting lower usage of the platform itself. This low usage of Facebook among adolescents found in this study is consistent with the latest report from Pew Research Center [4].

Notably, perceived realism significantly moderated the effects of social media–based e-cigarette message exposure on attitude toward e-cigarette use (Hypothesis 3a). When perceived realism was high, the exposure effect on attitude was amplified, but when perceived realism was low, the exposure effect was mitigated. This pattern was consistent across the four platforms of Facebook, Instagram, Snapchat, and YouTube (Figure 2). These findings indicate that social media realism judgment can be an important variable to include in future intervention efforts to change attitude and prevent e-cigarette use among adolescents.

Importantly, the perceived realism scale of this study captures the unique aspect of social media content. The social media realism scale of this study taps into the user-generated content and how it reflects peer users’ true selves. In contrast, traditional mass media content comprises media professional–generated content. This difference can constitute an important conceptual distinction between (mass) media literacy [52] and social media literacy. Efforts to curb the harmful effects of social media on adolescents should be cognizant of this participative and user-generated aspect of the social media world and address the distinctiveness in the conceptualization and development of interventions.

The posited networked nature of “social” in social media was further demonstrated by the finding of a three-way interaction. Social norm moderated the effect of social learning motivation on the relationship between exposure and attitude (Hypothesis 3b). Among adolescents who did not have friends who used e-cigarette, the effect of social learning motivation was mitigated, but among those with friends who used e-cigarettes, the effect was amplified. These results suggest that research on the uses and effects of social media should consider the interpersonal and social contexts of social media use as well, incorporating both the online and offline networks, as adolescents’ risk exposure and risk behavior may occur at this interface. Likewise, future e-cigarette control efforts should address the online and offline social environments of adolescents as they interface.

The same pattern of three-way interaction, however, was not found for other social motivations including comparison and filter (Hypothesis 3c-d), suggesting that their roles may be more complex than those of social learning motivation. As these motivations can play a role in the effects of social media use, future research should continue to examine these motivations and their interplay with the composition, structure, and size of social networks of adolescents.

### Limitations and Suggestions for Future Research

Although the outcome variable of this study was attitude rather than behavior, meta-analyses have consistently demonstrated that attitude predicts behavior [53,54]. Moreover, as the occurrence of behavior can be contingent upon various structural factors that facilitate or hinder the behavior [55], this study’s focus on attitude provides an up-close look at the relationships among motivations, uses, and effects of social media.

Future research could increase the number of items in some of the dimensions (eg, social comparison and filter; Multimedia Appendix 1) and test the scale with different populations to clarify the factor structure. Research may also benefit by scrutinizing the properties and functions of some of the dimensions of the scale, including social comparison and filter dimensions, as the posited three-way interaction among these dimensions, exposure, and social norm was not observed. Finally, research should continue to investigate and identify the core attributes, features, or affordances that cut across existing and emergent social media platforms, to better understand their uses and effects.

### Implications for Theory and Practice

This study fills a gap in the literature on social media effects on e-cigarette use through its investigation of the mechanisms of social media effects on risk behavior likelihood among adolescents. Through the investigation, it identified motivations, mediators, and moderators of social media effects. The results show not only how the motivations and uses impact adolescents’ attitude toward e-cigarette use, but also how the harmful effects could be mitigated. These findings inform the theory of social media effects and intervention for preventing harmful social media effects.

This study provides specific practical implications for future intervention efforts. It suggests that understanding and addressing the motivations associated with social media use are important, as it found that differential motivations have differential impact in the process of social media effects on e-cigarette use. Furthermore, this study shows that understanding and addressing self-expression and social learning motivations will be especially important, as these motivations exerted the strongest influence on frequency of social media use, exposure to e-cigarette messages, and attitude toward e-cigarette use. For example, providing youth with creative ways to participate in health campaigns could be a way to channel their self-expression motivation. A recent study found that production of digital counter messages by youth helped reduce risk behavior among them [56].

Instagram and Snapchat emerged as two of the more consequential social media platforms for adolescents. The results of this study show that the greater impact of these two platforms may stem from their capacities to satisfy the self-expression and social learning motivations of adolescents. As noted above, channeling these motivations to healthy directions will be important in future research and action for e-cigarette control. Instagram, especially, is noteworthy, as its visual focus may accommodate the visual nature and images of e-cigarette use behavior and their diffusion on social media and networks. Coupled with its significant impact on adolescents found in this study, the visual impact of Instagram merits further research. Recent research found that Instagram serves self-expressive and social engagement functions and that differential risk beliefs and emotions activate differential engagement and social support [57].

A new construct—social media realism—emerged as a significant moderator of social media effects. Social media realism consistently moderated the effects of exposure to e-cigarette message on e-cigarette attitude across adolescents’ four most popular social media platforms. These results suggest that correcting social media realism is critical to addressing the harmful effects of social media. In light of its significant role identified in this study, future research should engage in in-depth investigation of social media realism, as this construct differs from past mass media–based conceptualizations and offers important directions for future interventions. This effort could be central to advancing the conceptual knowledge basis of social media literacy.

The three-way interaction among exposure, social learning motivation, and social norm indicates that efforts to reduce the effects of social media e-cigarette may not be fully efficacious if they do not take into account the social contexts of adolescents. As the moderating effect of social learning motivation is contingent upon the social normative environment of adolescents, intervention efforts should address both the online and offline social contexts of adolescent lives and risk vulnerability.

### Conclusions

In summary, this study contributes to conceptual knowledge about the process of social media effects on risk behavior attitudes. The motivations of social media use and their mediating and moderating roles identified in this study will inform future research.

## Appendix

Multimedia Appendix 1 Social Media Use Motivation Scale.

## Abbreviations

e-cigarette: electronic cigarette
GFI: goodness-of-fit index
CFI: comparative fit index
RMSEA: root mean square error of approximation
BIC: Bayesian information criterion
