# Supplementary figures and images for: Mechanisms of Social Media Effects on Attitudes Toward E-Cigarette Use: Motivations, Mediators, and Moderators in a National Survey of Adolescents
Source: J Med Internet Res. 2019 Jun 27;21(6):e14303. doi: 10.2196/14303 (PMC6620891; doi:10.2196/14303)

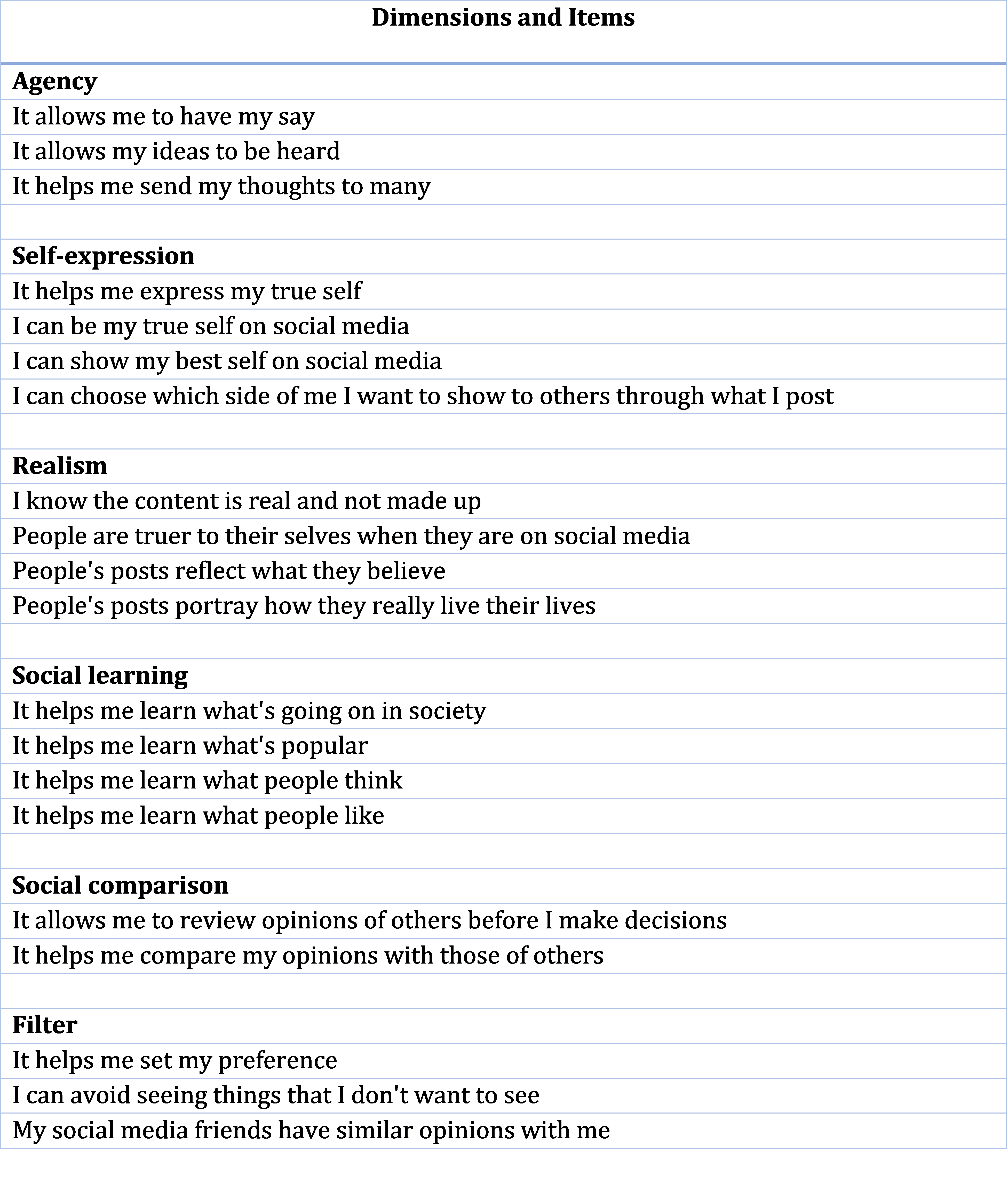

Supplement: Multimedia Appendix 1 [file jmir_v21i6e14303_app1.png]
